# Abstracts and Selections

**Published:** 1919-05

**Authors:** 


### Abstracts and Selections.

An interesting case of urogenital tuberculosis is reported by Claude G. Hofman in the American Journal of Surgery. With a positive history of pulmonary tuberculosis of four months standing, the patient deveoped pollakiuria, vesical tenesmus, etc., January, 1918, this continuing through February with a loss of seventeen pounds in weight. By March the 4th, 1918, he had developed acute urinary retention. His prostate was found so large that a satisfactory examination could not be made. During palpation of the prostate it seems an intracapsular abscess was ruptured discharging a little pus. This was examined and found to contain tubercular bacilii with other bacteria. A vaccine made from the material proved useful, for after the second injection the temperature became normal and he improved sufficiently to be discharged April the 20th. On June the first he returned, however, with a developed tuberculosis of the right testicle. Orchoidectomy was performed July 8. Improvement followed the operation with a gain of fourteen pounds. Cystoscopy, previously impossible, now revealed a tubercular bladder and urethral catheterization showed right kidney involvment. On October 2 epididymo-orchitis developed on the unaffected side (left) necessitating another operation. This was performed October 24. Improvement followed and has continued till the present day. The patient has gained six pounds. Dr. Hifman reports this case to show how rapidly urogenital tuberculosis develops and how pulmonary tuberculosis remains in a quiesent stage during this period.

---

Acid-forming bacteria as a remedy for Pruritus Ani is the latest suggestion offered for this most distressing symptom. Doctor J. H. Kellogg is the originator of the idea. His suggestion is that "an application of a special bacterial culture containing the Bacillus Bulgaricus, the B. Acidophilus, the streptococcus lactis, and the B. biffidus, all of which are active producers of lactic acid" be made following through cleansing with soap and water. His theory is that the bacteria which cause pruritus ani are unable to live in the presence of acid-forming bacteria. If his theory is correct, and it is sufficiently plausible to invite a testing of it, plain buttermilk, in the immediate absence of Bulgarian buttermilk, ought to give just as good results. Give it a trial.

